# Effects of Liquid Fructose Supplementation and Chronic Unpredictable Stress on Uterine Contractile Activity in Nonpregnant Rats

**DOI:** 10.3390/ijms25126770

**Published:** 2024-06-20

**Authors:** Zorana Oreščanin Dušić, Sanja Kovačević, Nataša Ristić, Danijela Vojnović Milutinović, Teodora Vidonja Uzelac, Duško Blagojević, Ana Djordjevic, Jelena Brkljačić

**Affiliations:** 1 Department of Physiology, Institute for Biological Research “Siniša Stanković”—National Institute of Republic of Serbia, University of Belgrade, 142 Despot Stefan Blvd, 11060 Belgrade, Serbia; zoranaor@ibiss.bg.ac.rs (Z.O.D.); teodora.vidonja@ibiss.bg.ac.rs (T.V.U.); dblagoje@ibiss.bg.ac.rs (D.B.); 2 Department of Biochemistry, Institute for Biological Research “Siniša Stanković”—National Institute of Republic of Serbia, University of Belgrade, 142 Despot Stefan Blvd, 11060 Belgrade, Serbia; sanja.kovacevic@ibiss.bg.ac.rs (S.K.); dvojnovic@ibiss.bg.ac.rs (D.V.M.); djordjevica@ibiss.bg.ac.rs (A.D.); 3 Department of Cytology, Institute for Biological Research “Siniša Stanković”—National Institute of Republic of Serbia, University of Belgrade, 142 Despot Stefan Blvd, 11060 Belgrade, Serbia; negicn@ibiss.bg.ac.rs

**Keywords:** fructose-fed rat, myometrium, oxytocin, adrenaline, oxidative stress

## Abstract

Increased fructose consumption and chronic stress, the major characteristics of modern lifestyle, impact human health; however, the consequences of their combination on the uterus remain understudied. In this study, we investigated contractile activity, morphology, and intracellular activity of antioxidant enzymes in uteri from virgin Wistar rats subjected to liquid fructose supplementation and/or unpredictable stress over 9 weeks. Contractile activity and uterine response to oxytocin or adrenaline were examined *ex vivo* using isolated bath chambers. Fructose supplementation, irrespective of stress, affected uterine morphology by increasing endometrium while decreasing myometrium volume density, attenuated uterine response to increasing doses of oxytocin, and increased glutathione peroxidase activity. Stress, irrespective of fructose, attenuated dose-dependent adrenaline-induced uterine relaxation. Stress, when applied solely, decreased mitochondrial superoxide dismutase activity. In the combined treatment, irregular estrous cycles and both reduced response to oxytocin and to adrenaline (as a consequence of fructose consumption and exposure to stress), along with fructose-related alteration of uterine morphology, were detected. In conclusion, fructose and stress affect uterine contractile activity, irrespective of each other, by inducing completely distinct responses in isolated uteri. In the combined treatment, the effects of both factors were evident, suggesting that the combination exerts more detrimental effects on the uterus than each factor individually.

## 1. Introduction

The modern way of living, which combines a sedentary lifestyle with unhealthy dietary habits and pervasive exposure to various stressors, is likely to contribute to a rising prevalence of various metabolic disorders, including obesity, metabolic syndrome, and type 2 diabetes [1,2,3,4,5]. In addition to impaired metabolic homeostasis, the impact of a modern lifestyle on female reproductive health is increasingly recognized [6,7].

The link between metabolic disorders, such as obesity and insulin resistance-related disorders, and impaired female fertility is well documented [6,8] and is best described for polycystic ovary syndrome [9]. However, unhealthy dietary habits might also impact female reproductive health even before the occurrence of metabolic pathophysiology [10]. Studies on animals have shown that both high-fructose and high-fat diets can trigger premature puberty onset, prolonged menstrual cycle, and impaired ovarian histology even without marked changes in body weight [11,12,13].

Girls and women of reproductive age are more frequently exposed to psychological stressors. Psychological stress is known to impair reproductive potential in females [14] through, among other things, hypothalamic-pituitary-gonadal axis dysfunction [15]. Exposure to stressors leads to an overactivation of the hypothalamic-pituitary-adrenal axis (HPA axis) and consequently to an increased release of cortisol (in humans) or corticosterone (in rodents). Activation of the HPA axis, acutely or chronically, has been shown to impair female reproduction either directly *via* the hypothalamus, pituitary, ovary, and uterus or indirectly *via* neuroendocrine pathways [14]. Stress interferes with ovarian follicle development [16] and ovulation [17] and impairs oocyte quality and embryo development [18]. 

The uterus is an often-overlooked tissue despite the fact that it is active throughout a woman’s life. Adequate uterine contractility is of importance for many reproductive functions, including sperm transportation, successful embryo transport and implantation, pregnancy, and parturition, while disrupted contractility may underlie some reproductive pathologies such as infertility, primary dysmenorrhea, and pregnancy complications (preterm delivery, caesarean section, spontaneous miscarriages, postpartum hemorrhage) [19]. Uterine contractility is precisely controlled by numerous mechanisms, including endocrine and neurogenic factors [19,20]. Additionally, subtle changes in the cellular redox status of the uterus were found to participate in the fine tuning of its contractility [21,22,23]. 

A considerable amount of knowledge related to uterine smooth muscle activity and reactivity to contractile and relaxant stimuli during specific physiological (pregnancy, parturition, menopause) and pathological conditions, including obesity, has been gathered [19,24,25,26]. Yet, the data related to modulations of myometrial contractile activity in relation to modern-lifestyle challenges are scarce and mostly focused on the pregnant rather than nonpregnant uterus. Investigation of these subtle changes in uterine contractility in the nonpregnant state is of importance, as these disturbances, which usually go under the radar, might be of relevance for a better understanding of clinical pathologies related to modern lifestyles, and may help in predicting responses to pharmacological treatments used in uterine contractility disorders or enable lifestyle interventions that can prevent reproductive health problems. 

Although increased fructose consumption and stress, as modifiable risk factors, often accompany each other in everyday life, the consequences of their combination are not fully understood, and the effects on the uterus remain understudied. We hypothesized that exposure to chronic unpredictable stress in combination with liquid fructose supplementation would induce more prominent effects on the physiological and histological features of the uterus than either of the factors separately. To test this hypothesis, we evaluated pharmacologically challenged contractile activity, morphometric, and histological features of the uterus and measured activity of antioxidant enzymes, cytosolic superoxide dismutase SOD1, mitochondrial superoxide dismutase (SOD2), catalase (CAT), glutathione peroxidase (GPx), and glutathione reductase (GR), in uteri from virgin female rats subjected to liquid fructose supplementation and/or chronic unpredictable stress. 

## 2. Results

### 2.1. Effects of Liquid Fructose Supplementation and/or Stress on Estrous Cycles Length and Regularity

All animals, except those subjected to both liquid fructose supplementation and stress, showed a regular estrous cycle of a normal length as observed in controls. In contrast, daily vaginal smear tests showed irregular estrous cycles with a prolonged diestrus stage in the experimental group subjected to the combined treatment (Table 1).

### 2.2. Histological and Stereological Analysis

**Figure 1:**
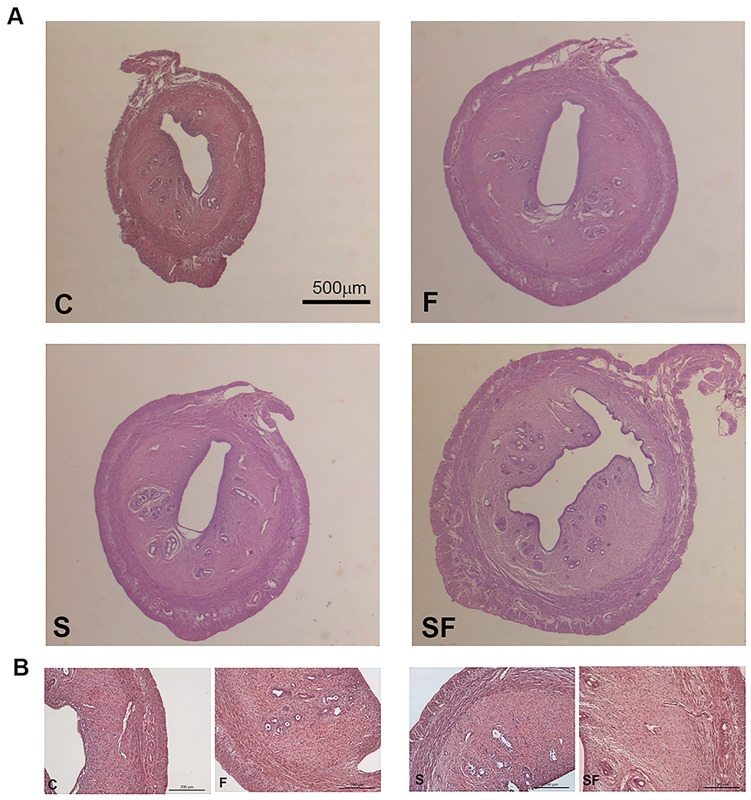
Hematoxylin–eosin-stained sections of uteri from control (C), fructose-fed (F), stress (S), and stress + fructose ss (SF) rats. Representative micrographs are shown. Panel (**A**); scale bar: 500 μm. Panel (**B**); scale bar 200 μm.

**Figure 2:**
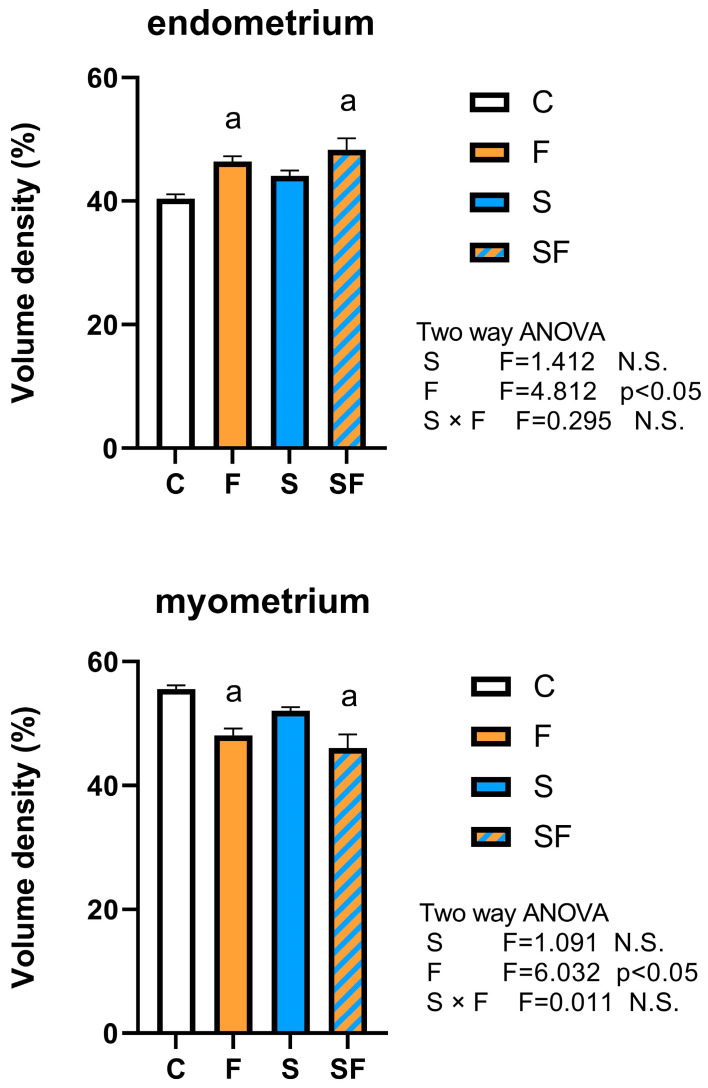
Morphometric and stereological analysis of uterus. Volume density of endometrium, myometrium, and perimetrium of control (C), fructose (F), stress (S), and stress + fructose (SF) groups. Data are expressed as mean ± SEM (n = 6). Two-way ANOVA was used to evaluate the effects of fructose and stress, and their interaction. Letter a denotes statistically significant main effect of fructose. *p* < 0.05 was considered statistically significant. N.S.—not significant.

The uterine tissue of the control animals had a typical appearance, with endometrium, myometrium, and perimetrium (Figure 1). The endometrium was well defined and consisted of a single layer of cuboidal epithelial cells lining the irregularly shaped lumen and endometrial stroma in which endometrial glands could be observed. The same histological organization was observed in the S, F, and SF experimental groups (Figure 1), but an increase in the thickness of the endometrium was observed in the F and SF groups, and this observation was subsequently confirmed by stereological analysis (Figure 2).

Two-way ANOVA showed a main effect of fructose on the volume density of endometrium (two-way ANOVA, F = 4.81, *p* < 0.05) and myometrium (two-way ANOVA, F = 6.03, *p* < 0.05). Increased volume density of endometrium along with decreased volume density of the myometrium were observed in both fructose-fed groups (Figure 2). 

### 2.3. Effects of Liquid Fructose Supplementation and/or Stress on Antioxidant Enzymes Activity

**Figure 3:**
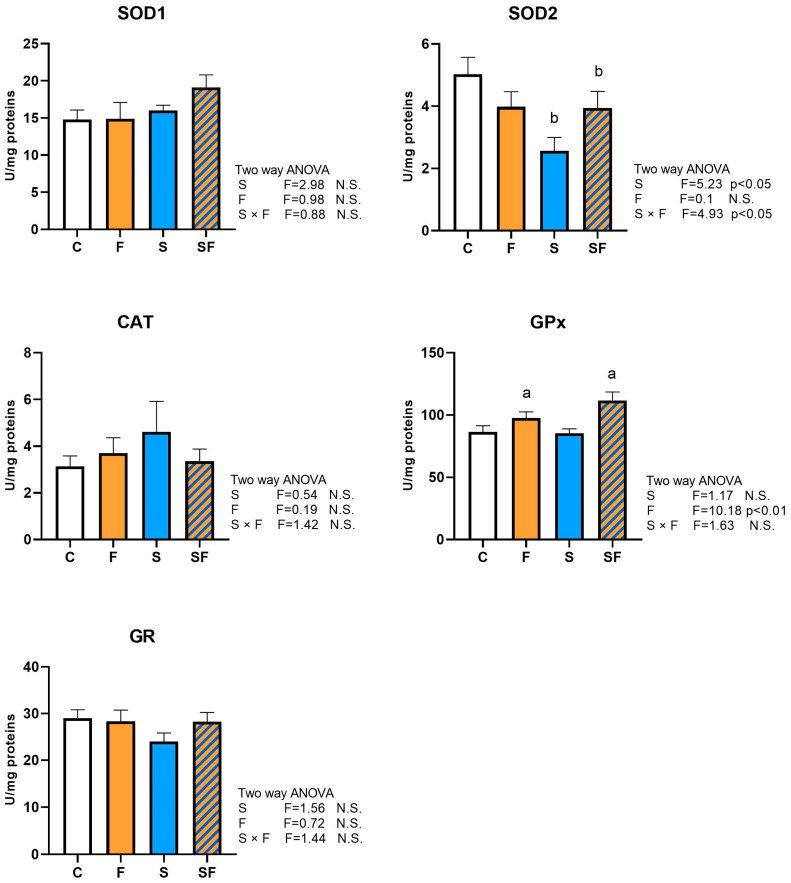
Effects of liquid fructose supplementation and/or stress on the activity of antioxidant enzymes. Groups: control (C), fructose-fed (F), stress (S), and stress + fructose (SF). Enzyme activity of SOD1, SOD2, CAT, GPx, and GR, in uterine whole cell extracts was measured spectrophotometrically. Bar graphs represent the mean ± SEM (*n* = 6 animals/group). Two-way ANOVA was used to evaluate the effects of fructose and stress and their interaction. Letters a and b denote statistically significant main effects of fructose and stress, respectively. *p* < 0.05 was considered statistically significant. N.S.—not significant.

The effect of chronic unpredictable stress on SOD2 activity (two-way ANOVA, F = 5.23, *p* < 0.05) and S × F interaction (F = 4.93, *p* < 0.05) was observed. *Post hoc* testing revealed a significant decrease in SOD2 activity in the group exposed solely to stress, as compared to the control group (*post hoc* Tukey HSD *t*-test, *p* < 0.05). At the same time, liquid fructose supplementation increased GPx activity (two-way ANOVA F = 10.18, *p* < 0.01) (Figure 3). The activities of SOD1, CAT, and GR were not affected by any of the treatments.

### 2.4. Effects of Liquid Fructose Supplementation and/or Stress on Uterine Contractility 

#### 2.4.1. Effects of Adrenaline on Phasic and Tone Uterine Contractions

**Figure 4:**
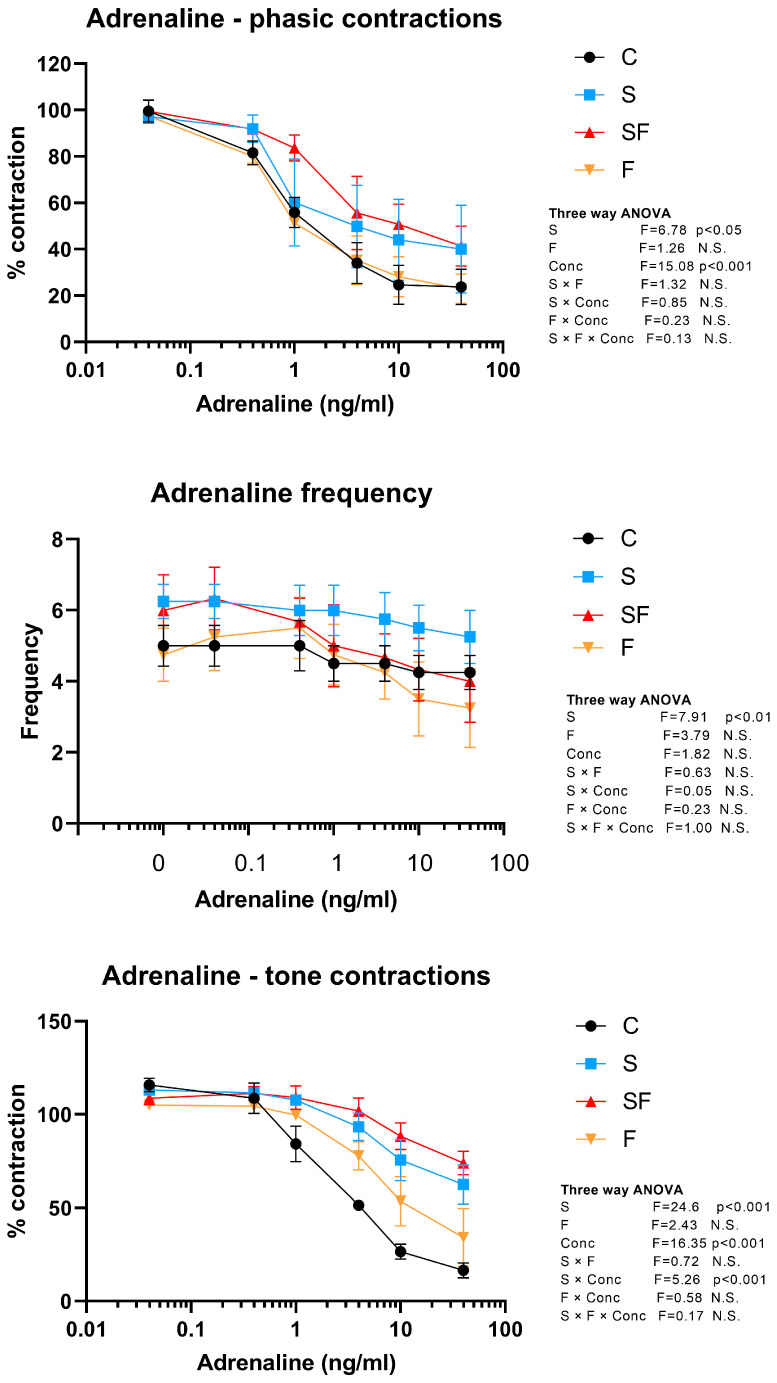
Effects of adrenaline on phasic and tone contractile activity of rat uterus in control (C), fructose (F), stress (S), and stress + fructose (SF) groups. Amplitudes, both for phasic and tonic contractions, were calculated as percentage of controls and expressed as mean values ± SEM (*n* = 6). Frequencies of phasic contractions are presented as number of contractions/5 min. Statistical significance was tested by three-way ANOVA (factors: fructose—F, stress—S, and adrenaline concentration—Conc), *p* < 0.05 considered statistically significant; N.S.—non-significant.

Adrenaline induced dose-dependent inhibition of the amplitude of phasic uterine contractions (three-way ANOVA, concentration effect, F = 15.08, *p* < 0.001). However, adrenaline was less effective in uteri from animals subjected to chronic unpredictable stress (three-way ANOVA, stress effect, F = 6.78, *p* < 0.05) (Figure 4). Frequency of contractions was increased in stressed animals (three-way ANOVA, stress effect, F = 7.91, *p* < 0.01) (Figure 4). Adrenaline also inhibited tone uterine contractions in a dose-dependent manner (three-way ANOVA, concentration effect, F = 16.35, *p* < 0.001). The response to adrenaline was significantly lower in the stressed animals (three-way ANOVA, stress effect, F = 24.6, *p* < 0.001), which was also confirmed by reduced response to increasing doses of adrenaline in the stressed animals (three-way ANOVA, S × Conc interaction, F = 5.26, *p* < 0.001) (Figure 4). 

#### 2.4.2. Effects of Oxytocin on Phasic Uterine Contractions

**Figure 5:**
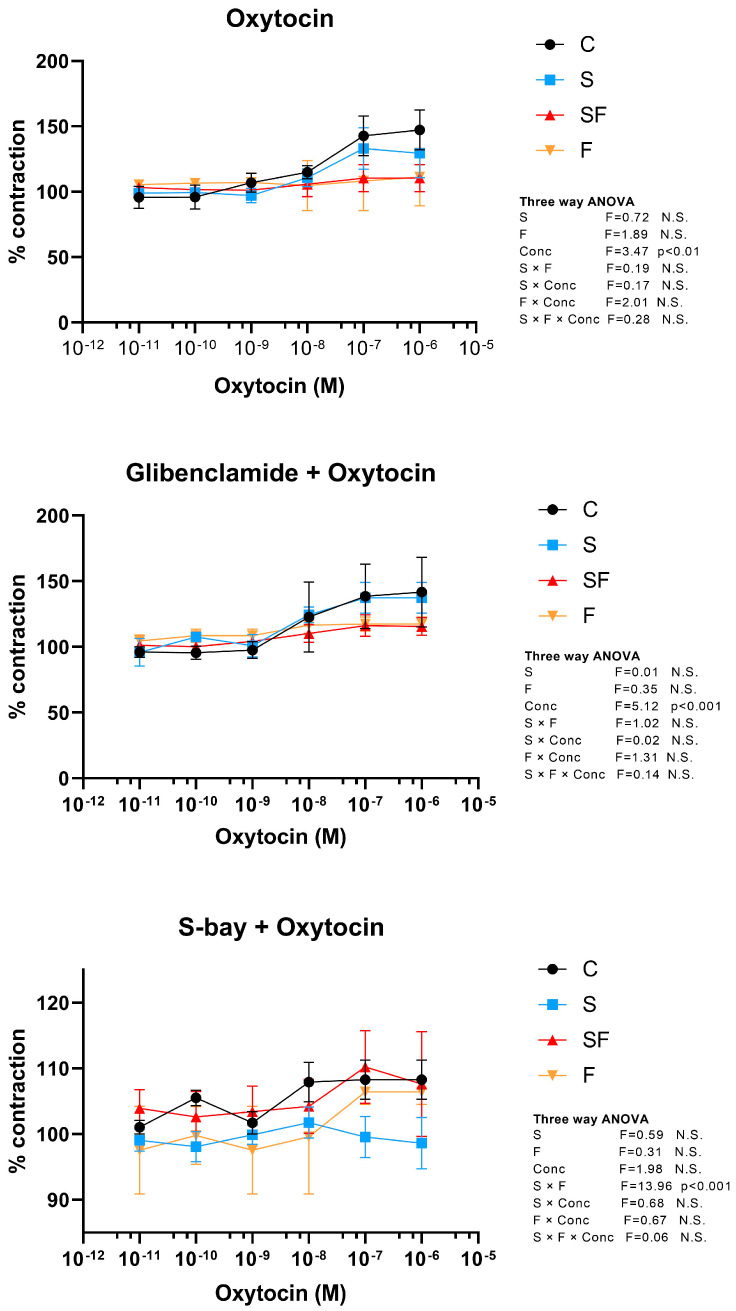
Effect of oxytocin alone and in the presence of glibenclamide or S-bay on phasic contractile activity of rat uterus in control (C), fructose (F), stress (S), and stress + fructose (SF) groups. Amplitudes were calculated as percentage of controls and expressed as mean values ± SEM (*n* = 6). Statistical significance was tested by three-way ANOVA (factors: fructose—F, stress—S, and oxytocin concentration—Conc), *p* < 0.05 considered statistically significant; N.S.—non-significant.

Oxytocin induced dose-dependent excitation of uterine contractility (three-way ANOVA, concentration effect, F = 3.47, *p* < 0.01), where concentrations of both 10^−7^ and 10^−6^ M of oxytocin induced a significant increase (*post hoc* Tukey HSD *t*-test, *p* < 0.05 for concentration effect) (Figure 5).

Pretreatment with glibenclamide had no effect on oxytocin treated uterus since three-way ANOVA again showed a concentration-dependent increase of uterine contractility (concentration effect, F = 5.12, *p* < 0.001), where doses of 10^−7^ and 10^−6^ M significantly increased uterine activity (*post hoc* Tukey HSD *t*-test, *p* < 0.01 for concentration effect) (Figure 5). 

Pretreatment with S-bay diminished oxytocin-induced elevation of contractile activity (three-way ANOVA, no concentration effect) but showed significant S × F interaction (three-way ANOVA, interaction S × F effect, F = 13.96, *p* < 0.001), which shows that inhibition of oxytocin-related elevation of contractile activity was more pronounced in the stressed animals (*post hoc* Tukey HSD *t*-test, *p* < 0.01) (Figure 5). 

**Figure 6:**
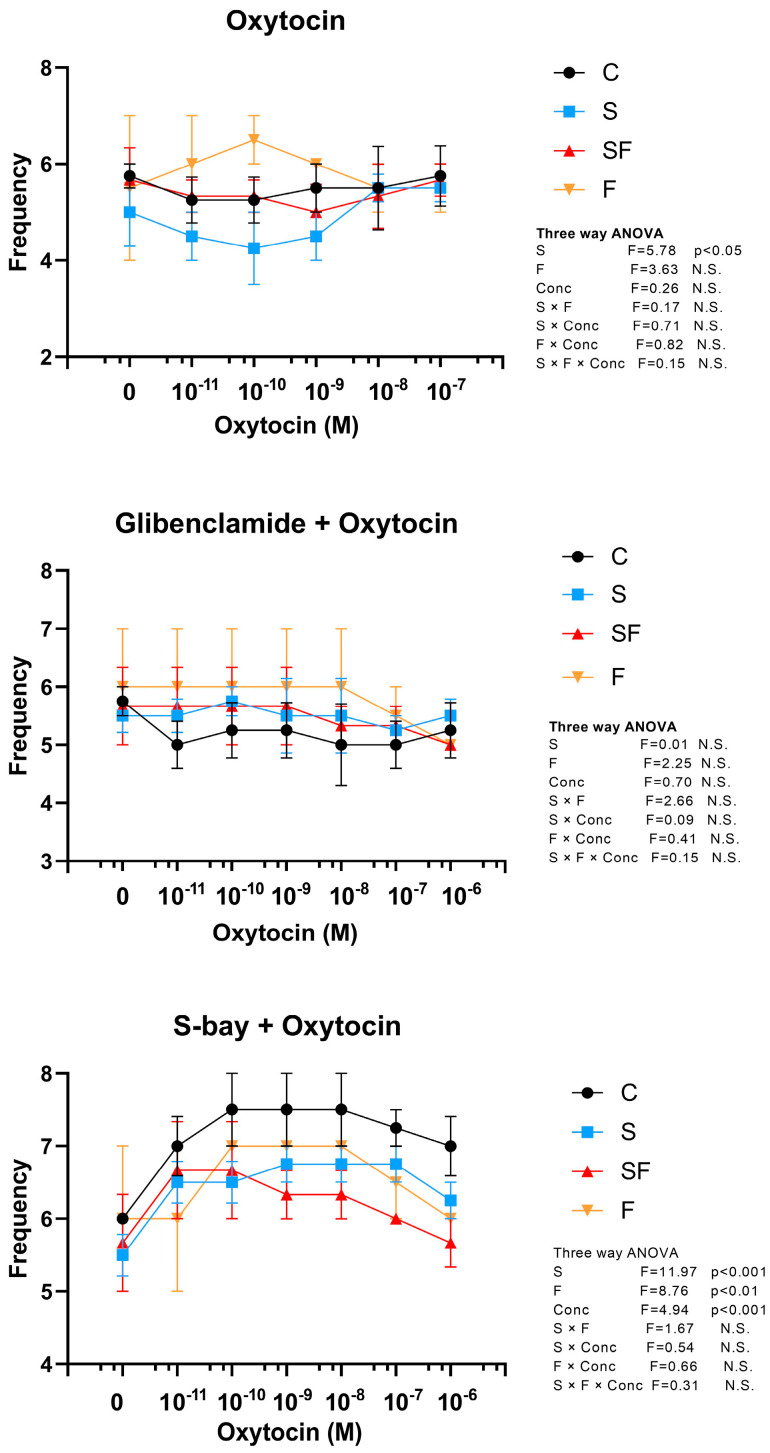
Effect of oxytocin alone and in the presence of glibenclamide and S-bay on phasic contractile activity of rat uterus in control (C), fructose (F), stress (S), and stress + fructose (SF) groups. Frequencies of phasic contractions are presented as number of contractions/5 min and expressed as mean values ± SEM (*n* = 6). Statistical significance was tested by three-way ANOVA (factors: fructose—F, stress—S, and oxytocin concentration—Conc), *p* < 0.05 considered statistically significant; N.S.—non-significant.

Oxytocin in the dose applied in our experiment did not affect frequency of contractions (three-way ANOVA, no concentration effect). However, stressed animals showed a weak decrease in uterine frequency during oxytocin treatment (three-way ANOVA, stress effect, F = 5.78, *p* < 0.05) (Figure 6). Oxytocin treatment in the presence of glibenclamide also showed no effect on uterine frequency in all groups (three-way ANOVA, no significant effect) (Figure 6). Pretreatment with S-bay and subsequent treatment with elevated oxytocin concentrations showed that dose of 10^−11^ M of oxytocin in the presence of S-bay elevated frequency, which remained at that level until the end of treatment (application of dose of 10^−6^ M of oxytocin) (three-way ANOVA, concentration effect, F = 4.94, *p* < 0.001, *post hoc Tukey t*-test, *p* < 0.01) (Figure 6). However, three-way ANOVA analysis also showed a significant effect of stress (F = 11.97, *p* < 0.001) on frequency, evidenced as reduced frequency in stressed animals. Furthermore, fructose-fed animals had lower frequency than their counterparts (three-way ANOVA, fructose effect, F = 8.76, *p* < 0.01) (Figure 6).

## 3. Discussion

Increased fructose consumption and stress are commonly encountered in everyday life and usually go hand-in-hand, yet the consequences of their combination on female reproductive health remain understudied. The current results show distinct effects of fructose and stress on uterine histology, contractile activity, and redox homeostasis, indicating that the combination of liquid fructose supplementation with chronic stress exerts more detrimental effects on the uterus than each factor individually. 

In the current study, an irregular cycle with prolonged diestrus stage was observed only in animals exposed to a combination of liquid fructose supplementation and stress, while stress and fructose separately had no effect on estrous cycle. Exposure to psychological stressors was associated with increase in cycle length, due to the prolonged diestrus [27]. The chronic unpredictable stress model may be used to assess the effects of chronic stress on female reproductive function, as it was found to induce psychological stress, decrease body weight, decrease ovarian reserve in female rats, prolong estrous cycle (primarily manifested as continuous diestrus), and alter serum levels of gonadotropin releasing hormone, follicle-stimulating hormone, estradiol, and anti-Müllerian hormone [28]. Also, metabolic disorders such as obesity, as well as unhealthy dietary habits in terms of unbalanced quality and quantity of nutrients, were found to affect estrous cycle. Namely, high fructose corn syrup was shown to induce obesity and adiposity in experimental animals and lengthen estrous cycle due to prolonged estrus [29]. As already mentioned, in our study, none of the separate treatments significantly prolonged estrous cycle, while only the combination induced continuous diestrus. Besides differences in the applied fructose dose and duration of the treatment, applied stressors, and strain of experimental animals, the inconsistency in the literature data might also be related to the presence/absence of obesity. Namely, we have previously reported in the same animal model that 9-week supplementation with 10% fructose solution did not induce obesity, while stress alone led to a decrease in body mass [30]. Although Ko et al. reported that high-fructose corn syrup extended the length of estrous cycle with prolonged estrus and diestrus stages and altered uterine morphology [13], our histological analyses showed that none of the treatments led to histopathological changes in the uterus. However, histological and stereological evaluations indicate that increased fructose consumption (alone or in combination with stress) affects uterine morphophysiology by increasing volume density of the endometrium and decreasing the volume density of the myometrium, whereas stress alone has no effect.

Precise and adequate uterine contractile activity is important for many reproductive functions. Uterine contractility is known to be associated with the female hormone cycle and uterine functionality [31,32]. It acts to peel endometrium during the menstrual period, influences sperm transport, nesting, embryo implantation, and pregnancy maintenance during the periovulatory phase, and is implicated in dysmenorrhea [31,33,34]. In the current study, oxytocin induced dose-dependent excitation of uterine contractility that was not mediated by K_ATP_ channels, since pretreatment with glibenclamide had no effect on oxytocin-induced contractions. On the other hand, uteri from both fructose fed groups were slightly refractory to oxytocin, as evidenced by the lower amplitude of the contractions both with and without pretreatment with glibenclamide. The fact that cholesterol directly inhibits myometrial contraction [35], along with our previously reported results that liquid fructose intake increases plasma cholesterol levels [36], may suggest hypercholesterolemia as one of the possible explanations for the impaired contractions in the fructose-fed groups. Also, Jie et al. have reported that cholesterol enrichment profoundly inhibits contraction force occurring spontaneously or in response to oxytocin, in a dose-dependent manner. This inhibitory effect on contractions and their associated Ca^2+^ transients was observed in both nonpregnant and pregnant laboring and nonlaboring myometrium [35]. Also, the observed decline in oxytocin-induced contractions in both fructose-fed groups could be related to thinner myometrium. 

S-bay K8644 is a potent L-type calcium channel agonist that has a positive inotropic effect resulting from increased calcium-induced calcium release from the sarcoplasmic reticulum [20]. Pretreatment with S-bay diminished oxytocin-induced elevation of contractile activity in control and stressed animals, as evidenced by a decline from 130% to 105% in control animals and to 95% in animals exposed solely to stress. This effect was not observed in fructose-fed groups, as they were already refractory to oxytocin. It seems that pre-opening of L-type calcium channels by S-Bay abolished the effects of external oxytocin and ceased its uterotonic activity in the control and stressed groups. Considering the effects on frequency, stress caused a small transitory reduction of frequency at low doses of oxytocin but not at high doses of oxytocin. At the same time, pretreatment with S-bay raised the frequency of contractions in the control and stressed animals. 

Chronic unpredictable stress altered the sensitivity of the uterus to external adrenaline. Namely, both phasic and tone contractions, as well as their frequency, were affected by stress. These disturbances of uterine functionality were neither mediated nor compensated by fructose. Although adrenaline at low concentrations inhibits uterine activity by reducing contractile force and uterine tone and disrupting the rhythmicity of contraction [37], in our study, animals exposed to stress showed a significant degree of insensitivity to the relaxant effect of adrenaline. Since the uterus exhibits tone and phasic contractions without significant nerve control [38], the observed effects can be related to the levels of adrenaline receptors in the uterus. Both types of uterine contractions have their roles in labor, faith of placenta, and bleeding. Therefore, chronic unpredictable stress could have serious detrimental consequences on uterine functionality. The weaker *ex vivo* response to adrenaline could be explained by the fact that stressed animals have already been repeatedly exposed to adrenaline, and this frequent exposure could attenuate their sensitivity. Further studies along these lines could shed more light on the underlying molecular mechanisms. 

Disturbances in antioxidant enzymes activity and the concomitant alterations in redox homeostasis might also contribute to altered uterine contractility. Both chronic stress and fructose were previously found to alter the redox status of the cell [39,40,41,42,43,44,45,46,47,48]. Our results confirmed that fructose-fed animals had higher uterine GPx activity, while stress alone led to a decrease in SOD2 activity. In line with this, Sadowska et al. [49] reported that variable sucrose administration caused an increase in GPx and a decrease in total SOD activities in the rat uterus. Fructose is a highly lipogenic sugar, and GPx could be involved in reduction of lipid hydroperoxides and hydrogen peroxide. When applied alone, stress led to a significant decline in SOD2 activity, while fructose attenuated stress-related decrease in SOD2 activity. The link between fructose consumption and oxidative stress appears to be rather complex, and fructose and its phosphorylated forms were shown to efficiently scavenge free radicals [50,51]. Fructose appears to act both prooxidatively and antioxidatively, depending on the dose, duration of the treatment, and pathophysiological milieu [52].

Taken together, the combined treatment induced irregular estrous cycle, manifested as continuous diestrus. Also, fructose-related alterations in uterus morphology, as well as altered response to oxytocin, were observed in animals subjected to the combined treatment. At the same time, a reduced response to adrenaline as a consequence of chronic unpredictable stress was observed. The chronic unpredictable stress paradigm used in our study to mimic everyday human life affects the redox state of the uterus and the response to adrenaline. Administration of liquid fructose, chosen to resemble a daily intake of sweet beverages, affects the physiological, histological, and redox properties of the uterus. The absence of obesity and adiposity allowed us to investigate the effects of fructose supplementation on reproductive health under stressful conditions and to determine whether the impairment in reproductive potential was due to fructose metabolism, independent of obesity. Our results indicate that unhealthy dietary habits in terms of unbalanced quality and quantity of nutrients affect female reproductive health, suggesting that reproductive disorders may precede the development of obesity. Furthermore, these disorders were exacerbated by stress. Therefore, lifestyle and nutritional interventions may represent a promising strategy to prevent or treat clinical problems related to female reproductive health caused by an unhealthy lifestyle. Further studies in this direction are required, as the results obtained in animals should be interpreted with caution when extrapolating to humans.

In conclusion, fructose and unpredictable stress induce metabolic and physiological changes that can impair the normal functionality and reproductive potential of female animals. Importantly, the combination of fructose and stress has more detrimental effects than either treatment alone. The intake of liquid fructose affects the morphology of the uterus and alters the uterine response to oxytocin, independent of stress, while stress, independent of fructose, attenuates the relaxing effect of adrenaline. In the combined treatment, a reduced response to both oxytocin and adrenaline (as a result of fructose consumption and stress) is observed, together with an irregular cycle that manifests as continuous diestrus.

## 4. Materials and Methods

### 4.1. Animals and Treatment

Female virgin Wistar rats (2.5 months old), bred in our laboratory, were randomly divided into four experimental groups (*n* = 6 rats/group): control (C), fructose (F), stress (S), and stress + fructose (SF). Allocation of the animals to the experimental groups was performed by an appropriate randomization method in order to ensure blinding and reduction of systematic differences in characteristics of animals assigned to experimental groups. Animals were kept under standard conditions, at 22 °C with a 12-h light/dark cycle. All rats were fed *ad libitum* with commercial rat food (Laboratory Rat Food R20: 20% protein, 62.6% carbohydrate and 3.2% fat, mineral and vitamin mix; Veterinary Institute Subotica, Serbia), as described previously [36]. F and SF groups had 10% (*w*/*v*) fructose solution instead of drinking water. Fructose concentration was chosen to resemble modern human lifestyle [53]. S and SF groups were subjected to a chronic unpredictable stress protocol (modified from [54]), which consisted of following daily stressors: forced swimming in cold water for 10 min, physical restraint for 60 min, exposure to a cold room (4 °C) for 50 min, wet bedding for 4 h, switching cages for 2 h, rocking cages for 1 h, and cage tilt (45°) overnight. The unpredictability of the stressors (type of daily stressor(s), number of stressor(s) (1 or 2), and the onset of stress exposure (between 9 a.m. and 4 p.m.)) was achieved through random selection at the beginning of the treatment. A particular stressor was never applied on two consecutive days or twice per day. Both fructose feeding and stress lasted for 9 weeks. 

All animal procedures were in compliance with Directive 2010/63/EU on the protection of animals used for experimental and other scientific purposes and were approved by the Ethical Committee for the Use of Laboratory Animals of the Institute for Biological Research “Siniša Stanković”, University of Belgrade (No. 02-11/14). 

### 4.2. Tissue Preparation

Animals were sacrificed in the diestrus phase of the estrous cycle by rapid decapitation. The stage of the estrous cycle was determined by examination of daily vaginal smears [55]. After decapitation, the uterine horns were rapidly excised and carefully cleaned of all fat and surrounding connective tissue, rinsed in De Jalon’s solution or Krebs Henseleit (KH), and splatted. To standardize sample collection, uterine strips for isolated organ bath studies were taken from the same region of the right uterine horn in all animals. The left uterine horn was divided, and one part was immediately frozen in liquid nitrogen and stored at −80 °C until further analysis of antioxidant enzymes activity, while the other part was fixed in paraformaldehyde for 24 h, dehydrated in ethanol of increasing concentration, enlightened in xylene, and embedded in paraffin wax (Histolab Product AB, Göteborg, Sweden). Each paraffin tissue block was serially sectioned at 5 µm thickness using a rotary microtome (RM2125RT Leica, Glostrup, Denmark) and processed for hematoxylin–eosin staining.

### 4.3. Isolated Organ Bath Studies

Uteri were mounted vertically in 10 mL volume organ baths with one end tied to a tissue holder and the other to a wire connected to a force transducer (Experimetria, Budapest, Hungary). The chambers containing De Jalon’s solution (in g/l: NaCl 9.0, KCl 0.42, NaHCO_3_ 0.5, CaCl_2_ 0.06, and glucose 0.5) or KH isotonic solution (118 mM NaCl; 4.7 mM KCl; 25 mM NaHCO_3_; 1.2 mM KH_2_PO_4_; 1.25 mM CaCl_2_; 1.2 mM MgSO_4_; 11 mM glucose (pH 7.4)) were maintained at 37 °C, aerated with a gas mixture of 95% oxygen and 5% carbon dioxide. Uteri were allowed to stabilize for 30 min at 1 g tension until a stable resting tone was acquired. Adrenaline effect on uterine contractility was studied on spontaneously active uteri placed in KH solution and on uteri placed in De Jalon solution treated with KCl (75 mM) to induce tone contractions. Both groups were treated with cumulative doses of adrenaline (concentrations: 0.04, 0.4, 1, 4, 10, 40 ng/mL). The effect of oxytocin on uterine contractility was studied on uteri placed in KH solution to achieve stable spontaneous contractions by cumulative oxytocin addition (concentrations: 10^−11^–10^−6^ M). Additionally, two sets of uteri treated with cumulative doses of oxytocin were pretreated with antagonist of K_ATP_ channels, glibenclamide (2 µM), or with activator of L-type Ca^2+^-channels, S-bay (0.1 µM). Changes in isometric force were recorded on a TSZ-04-E Tissue Bath System (Experimetria) (MDE Research, Heidelberg, Germany).

### 4.4. Antioxidant Enzymes Activity 

Antioxidant enzymes activity was determined in the whole cell extracts, as previously described [23]. Thawed uteri were homogenized and sonicated in 0.25 M sucrose, 1 mM, ethylenediaminetetraacetic acid, and 0.05 M TrisHCl buffer (pH 7.4) before centrifugation for 90 min at 105,000× *g*. The supernatants were used to measure SOD1, SOD2, CAT, GPx, and GR activities spectrophotometrically. Enzyme activities are expressed as units (U) per mg of protein, as described previously [56]. All measurements of absorbance were performed using a Shimadzu UV-160 spectrophotometer (Shimadzu Scientific Instruments, Shimadzu Corporation, Kyoto, Japan).

### 4.5. Histological and Stereological Analysis

All analyses were performed using a workstation comprised of a microscope (Olympus BX-51, Tokyo, Japan) equipped with a microcator (Heidenhain MT1201, San Jose, CA, USA), a motorized stage (Prior Scientific, Cambridge, UK), and a CCD video camera (PixeLink, Ottawa, ON, Canada). The main objectives were a planachromatic 10× dry lens and 20× dry lens. Control of stage movements and interactive test grids and unbiased dissector frames were provided by the newCAST stereological software package (Visiopharm, Hørsholm, Denmark).

The volume density of the main uterine compartments (endometrium, myometrium, and perimetrium) was determined according to the Cavalieri principle [57,58] using the newCAST stereological software package Visiopharm Integrator System (VIS) version 3.2.7.0 (Visiopharm; Denmark). Sampling of 5 µm uterine sections was performed systematically, uniformly at random. Every 20th uterine section from each of the tissue blocks was analyzed stereologically and morphometrically. The first section to be analyzed was randomly selected using a table of random numbers. 

### 4.6. Data Presentation and Analysis

Data are presented as means ± SEM. Effects of fructose and stress, and their interaction on morphometric parameters and the activity of antioxidant enzymes, were analyzed by two-way analysis of variance—ANOVA (factors fructose feeding—yes/no or stress—yes/no), followed by Tukey’s HSD post hoc test (significance: *p* < 0.05). The effects on contractility were analyzed by three-way ANOVA (concentration as an additional factor along with fructose and stress). Statistical analysis was performed using STATISTICA 8.0 software (StatSoft, Inc., Tulsa, OK, USA). A probability level of *p* < 0.05 was considered statistically significant.

## Figures and Tables

**Table 1:** Effects of liquid fructose supplementation and/or stress on estrous cycle.

	Control	Fructose	Stress	Stress + Fructose
Duration of the estrous cycle (days)	5 ± 1.7	5 ± 2.2	5 ± 0.7	* n.d. (prolonged diestrus)
Females with irregular cycles	0/6	1/6	1/6	5/6

The vaginal smears were taken daily over the last 18 days of the treatment. Values are expressed as mean ± SEM. * not determined

## Data Availability

The original contributions presented in the study are included in the article, further inquiries can be directed to the corresponding author.
